# Testing the combined effects of probiotics and prebiotics against neurotoxic effects of propionic acid orally administered to rat pups

**DOI:** 10.1002/fsn3.2418

**Published:** 2021-06-23

**Authors:** Anwar Al Suhaibani, Abir Ben Bacha, Mona Alonazi, Ramesa Shafi Bhat, Afaf El‐Ansary

**Affiliations:** ^1^ Biochemistry Department Science College King Saud University Riyadh Saudi Arabia; ^2^ Laboratory of Plant Biotechnology Applied to Crop Improvement Faculty of Science of Sfax University of Sfax Sfax Tunisia; ^3^ Central Laboratory King Saud University Riyadh Saudi Arabia

**Keywords:** bee pollen, oxidative stress, neuroinflammation, probiotics, propionic acid

## Abstract

The present study investigated the combined effects of mixed probiotic and bee pollen on brain intoxication induced by propionic acid (PPA) in rat pups. Thirty western albino rats were divided into five groups, six animals each: (1) Control group receiving phosphate‐buffered saline; (2) Probiotic and bee pollen‐treated group being administered at the same dose with 200 mg/kg body weight; (c) PPA‐treated group receiving a neurotoxic dose 250 mg/kg body weight of PPA for 3 days; (d) Therapeutic group being administered the neurotoxic dose of PPA followed by probiotic and bee pollen treatment 200 mg/kg body weight; (e) Protective group receiving probiotic and bee pollen mixture treatment followed by neurotoxic dose of PPA. Selected biochemical parameters linked to oxidative stress, energy metabolism, and neurotransmission were investigated in brain homogenates from all the five groups. PPA treatment showed an increase in oxidative stress markers like lipid peroxidation coupled with a significant decrease in glutathione level. Impaired energy metabolism was ascertained via the alteration of creatine kinase (CK) and lactate dehydrogenase (LDH) activities. Dramatic increase of Na^+^ and K^+^ concentrations together with a decrease of GABA and IL‐6 and an elevation of glutamate levels in PPA‐treated rat's pups confirmed the neurotoxicity effect of PPA. Interestingly, the mixed probiotic and bee pollen treatment were effective in restoring the levels of glutamate, GABA, and IL‐6 in addition to normalizing the levels of lipid peroxidation and glutathione and the activities of CK and LDH. The present study indicates that mixed probiotic and bee pollen treatment can improve poor detoxification, oxidative stress, and neuroinflammation as mechanisms implicated in the etiology of autism.

## Graphical abstract

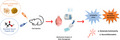

## 1 INTRODUCTION

Scientific research highlighted the effectiveness of antioxidants, vitamins, minerals, polyunsaturated fatty acids, prebiotics, and probiotics as inducer of optimistic effects on the body. Among these "active ingredients," the prebiotics play a mainly important role because of their ability to selectively stimulate the growth and/or the metabolic activity of one or more beneficial bacterial species in the host gut. Probiotics, on the other hand, as "live active ingredients" are food supplements that are made up of live microorganisms that react favorably on the host improving the intestinal microbial balance. The probiotic microorganisms mostly belong to *Lactobacillus* and *Bifidobacterium* families (Cagnasso et al., 2002; Prasad et al., 1998; Rubio et al., 2014).

*Lactobacillus* is the largest of lactate bacteria genera, comprising microaerophilic, no spore‐forming, catalase‐negative, and Gram‐positive bacteria. *Lactobacilli* are commonly found in various environment such as plants and soil, human, and animal mucosal surfaces as well as in dairy products (Lahtinen et al., 2011). Selection of potential probiotics requires numerous characteristics (Guo et al., 2010). In fact, the microorganism should be nonpathogenic and could tolerate the physiological concentrations of bile and the acidic pH in stomach and, therefore, to survive in the gastrointestinal tract; must present antagonistic activity against intestinal pathogens and should display desirable surface hydrophobicity for colonization (Mishra & Prasad, 2005).

It is all the more impressive to realize how some probiotics succeed in improving their "performance" by cooperating together. Combined effects between prebiotics and probiotics have been demonstrated to create the optimum substrate necessary for the growth of several bacteria species, in particular, Bifidobacterium which is well known to be lower in autism spectrum disorder (ASD) patients. Tomova and collaborators (2015) investigated the impact of mixed probiotic (Children Dophilus) administration for 4 months on gut microbiota (GM) composition in ASD patients. Authors were able to observe an increase in bifidobacterial numbers with a modulation of the *Bacteroidetes/Firmicutes* ratio (Tomova et al., 2015). Prebiotics as food ingredients are selectively metabolized by indigenous beneficial bacteria therefore positively modulating GM. However, their impacts are not well documented in autism.

MacFabe et al. (2007) and El‐Ansary et al. (2012) demonstrated that intraventricular infusion or oral administration of PPA can modify both behavior and brain in the laboratory animals in a way that is consistent with human ASD symptoms. The neuropathological, behavioral, and biochemical results in the MacFabe PPA model provide further support for the hypothesis that autism could be a systemic metabolic encephalopathic process affecting the brain. The similarities in oxidative stress and innate neuroinflammatory changes between their animal model and human ASD cases could exhibit comparable immune metabolic or mediated processes (Moritz & Ayus, 2010) indirectly or directly linked to PPA. Particularly are their observations of broad alterations in lipid peroxides and glutathione (GSH) levels which may provide a common mechanism for elevated environmental sensitivity to diverse environmental compounds and increased oxidative stress (Hiratani et al., 1997). El‐Ansary et al. (2017) evidenced the induction of imbalanced excitatory/inhibitory function in PPA rodent model. This information motivates our interest to test the combined effects of mixed probiotic and bee pollen in ameliorating the impaired biochemical features induced with PPA in rat pups through the measurement of K^+^, Na^+^, CK, LDH, interleukin‐6 (IL‐6), glutamate, ɣ‐ aminobutyric acid (GABA) in tissue homogenates of PPA intoxicated, and treated or protected with a symbiotic (probiotic+bee pollen).

## 2 MATERIAL AND METHODS

### 2.1 Animals

**FIGURE 1:**
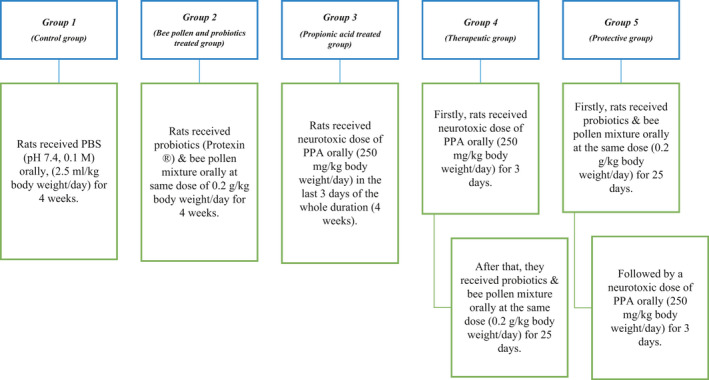
Schematic presentation of the animal model experimental design. Probiotics (PROTEXIN^®^), a product of Probiotics International Limited (UK). Bee pollen (NZ Bee Pollen Granules) was product of Happy Valley (New Zealand). Propionic acid was Sigma‐Aldrich (USA) product.

All animal experiments were approved by the Ethics Committee of the College of Science at King Saud University and were carried out according to the national guidelines for the use and care of animals. Thirty male Wister albino rats (60–70 g) were kept in cage (42.5 cm × 26.5 cm × 14.5 cm) under standard laboratory conditions (temperature 23°C, humidity 37%, and light for 12 hr) and the food supply was from Grain Silos and Flour mills organization. Rats were randomly divided into five groups of six animals as shown in Figure 1. Carbon dioxide‐anesthetized rats were decapitated at the end of feeding trials, and their brains were removed from the skull and dissected into small pieces. After homogenization in bidistilled water (1:10, w/v), brain samples were stored at −80°C.

### 2.2 Biochemical analyses

The activity of catalase was determined according to the method of Maehly and Chance (1954) by following the rate of hydrogen peroxide dissociation/minute by the catalase enzyme at 240 nm. LDH (REF 047, United Diagnostics Industry, Dammam‐KSA) and CK (REF 028, United Diagnostics Industry, Dammam‐KSA) activities were assessed using the methods described by Szasz et al. (1976) and Amador et al. (1963), respectively, by following the rate of NADH formation, which is directly proportional to the sample LDH or CK activity, at 340 nm.

The levels of GSH and lipid peroxidation in the brain samples were measured spectrophotometrically according to the protocols described by Beutler (1963) and Ruiz‐Larrea et al. (1994), respectively.

K^+^ (REF 10118) and Na^+^ (REF 573351) levels were determined using diagnostic kits from Human (Germany) while glutamate (REF KA1909, Abnova), GABA (REF ER1707, Fine Test), and IL‐6 (REF ER0042, Fine Test) were investigated using ELISA kits following the manufacturers' instructions. All measurements were performed in duplicate, and the mean of two different readings was calculated. Quality control assays were performed to evaluate experimental reproducibility through the inter‐ and intra‐assay coefficients of variability (%CV).

### 2.3 Statistical analysis

The statistical package for the social sciences (SPSS) was used to analyze the data of the current study. Obtained results are shown as mean ± standard deviation (S.D). Statistical correlations and comparisons between parameters were performed using Pearson's correlation coefficient (*r*) and the independent *t* test, respectively. To evaluate brain neurotoxicity in animal modeling, the receiver operating characteristics (ROC) curve and the area under the ROC curve (AUC) were used as a fundamental tool. Only *p* values ≤0.05 were considered significant.

## 3 RESULTS

**FIGURE 2:**
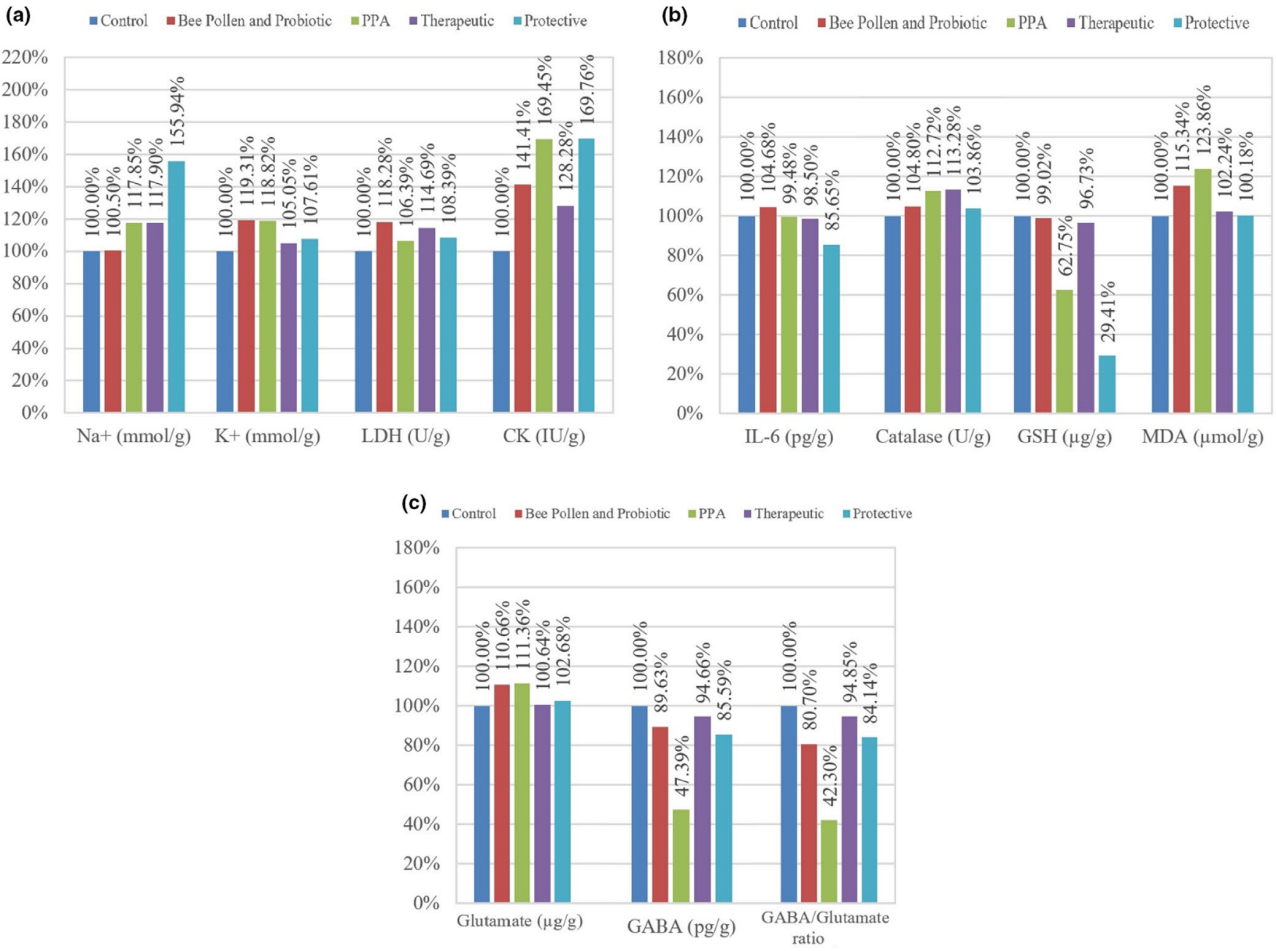
(a–c): Percentage change of all parameters measured in the brain homogenate of treated rats pups compared with control

Table 1 showed the brain homogenates levels of N^+^, K^+^, LDH, CK, IL‐6, GABA, GSH, lipid peroxidation, glutamate, catalase, and GABA/glutamate ratio in addition to their percentage change (also shown in Figure 2) relative to control of all the tested groups. Table 1 and Figure 2 revealed that PPA‐treated group exhibited a significant increase in CK (169.45%), a significant decrease of GABA (47.39%), GABA/glutamate ratio (42.3%) and GSH (62.75%) compared with control. The brain homogenate of bee pollen and probiotics‐treated rat pups also showed improvement in the tested parameters as shown in Table 1 and Figure 2. Pearson's correlations between the different measured parameters were performed and presented in Table 2 showing either positive or negative correlations and may clarify the inter‐relationship between different studied parameters as etiological mechanisms associated with neurotoxicity of the brain. ROC analysis is also presented in Table 3 showing AUC, specificity, and sensitivity of all measured parameters.

**TABLE 1:** Mean ± *SD* of all the measured parameters in brain homogenate of treated rats' pups compared with control group

Parameters	Groups	*N*	Min.	Max.	Mean ± *SD*	Percent change	*p* value^a^	*p* value^b^
Na^+^ (mmol/g)	Control	6	0.356	0.470	0.413 ± 0.044	100.00		.001
	PPA	6	0.399	0.519	0.487 ± 0.045	117.85	.035	
	Bee pollen and probiotics	5	0.389	0.445	0.415 ± 0.026	100.50	1.000	
	Therapeutic	5	0.374	0.548	0.487 ± 0.069	117.90	.045	
	Protective	4	0.617	0.670	0.644 ± 0.023	155.94	.001	
K^+^ (mmol/g)	Control	6	0.079	0.097	0.088 ± 0.007	100.00		.157
	PPA	6	0.095	0.130	0.104 ± 0.013	118.82	.128	
	Bee pollen and probiotics	5	0.088	0.148	0.105 ± 0.025	119.31	.139	
	Therapeutic	5	0.087	0.095	0.092 ± 0.003	105.05	.950	
	Protective	4	0.092	0.099	0.095 ± 0.003	107.61	.854	
LDH (U/g)	Control	6	21.49	41.46	30.80 ± 7.22	100.00		.461
	PPA	6	23.92	38.70	32.77 ± 5.35	106.39	.922	
	Bee pollen and probiotics	5	27.98	41.68	36.43 ± 5.32	118.28	.276	
	Therapeutic	5	29.50	41.46	35.32 ± 4.39	114.69	.460	
	Protective	4	32.91	33.88	33.38 ± 0.44	108.39	.873	
CK (IU/g)	Control	6	18.94	69.23	48.36 ± 18.04	100.00		.001
	PPA	6	76.64	93.64	81.95 ± 6.24	169.45	.001	
	Bee pollen and probiotics	5	40.16	82.81	68.39 ± 16.61	141.41	.054	
	Therapeutic	5	54.51	73.40	62.04 ± 7.43	128.28	.259	
	Protective	4	71.23	91.58	82.10 ± 8.40	169.76	.002	
IL‐6 (pg/g)	Control	6	487.23	658.60	564.94 ± 55.24	100.00		.062
	PPA	6	510.75	665.32	562.00 ± 56.86	99.48	1.000	
	Bee pollen and probiotics	5	477.15	656.92	591.40 ± 67.68	104.68	.818	
	Therapeutic	5	525.87	581.32	556.45 ± 19.86	98.50	.996	
	Protective	4	440.19	512.43	483.87 ± 34.57	85.65	.075	
Catalase (U/g)	Control	6	17.93	23.37	20.79 ± 1.95	100.00		.327
	PPA	6	20.08	30.22	23.44 ± 4.08	112.72	.259	
	Bee pollen and probiotics	5	20.47	22.75	21.79 ± 0.85	104.80	.922	
	Therapeutic	5	22.29	26.66	23.56 ± 1.85	113.28	.263	
	Protective	4	19.14	24.88	21.60 ± 2.55	103.86	.970	
GSH (µg/g)	Control	6	13.74	20.25	18.44 ± 2.58	100.00		.001
	PPA	6	7.96	15.91	11.57 ± 2.71	62.75	.001	
	Bee pollen and probiotics	5	15.19	21.70	18.26 ± 2.77	99.02	1.000	
	Therapeutic	5	14.47	23.87	17.84 ± 3.70	96.73	.990	
	Protective	4	3.62	7.96	5.43 ± 1.91	29.41	.001	
MDA (µmol/g)	Control	6	2.05E‐03	3.85E‐03	2.96E‐03 ± 6.98E‐04	100.00		.291
	PPA	6	2.82E‐03	4.36E‐03	3.67E‐03 ± 5.84E‐04	123.86	.218	
	Bee pollen and probiotics	5	3.21E‐03	3.72E‐03	3.41E‐03 ± 2.19E‐04	115.34	.622	
	Therapeutic	5	1.99E‐03	3.91E‐03	3.03E‐03 ± 7.70E‐04	102.24	.999	
	Protective	4	2.24E‐03	3.97E‐03	2.96E‐03 ± 8.38E‐04	100.18	1.000	
Glutamate (µg/g)	Control	6	675.78	895.31	796.60 ± 75.78	100.00		.137
	PPA	6	751.56	969.53	887.07 ± 75.11	111.36	.141	
	Bee pollen and probiotics	5	822.66	932.81	881.55 ± 40.48	110.66	.211	
	Therapeutic	5	717.19	950.78	801.72 ± 94.80	100.64	1.000	
	Protective	4	731.25	897.66	817.97 ± 68.27	102.68	.977	
GABA (pg/g)	Control	6	294.90	465.85	382.09 ± 57.40	100.00		.006
	PPA	6	84.80	232.81	181.09 ± 51.73	47.39	.003	
	Bee pollen and probiotics	5	219.53	468.97	342.45 ± 113.67	89.63	.876	
	Therapeutic	5	263.22	537.85	361.69 ± 107.49	94.66	.987	
	Protective	4	213.93	464.29	327.03 ± 105.56	85.59	.745	
GABA/Glutamate ratio	Control	6	3.78E‐07	6.19E‐07	4.85E‐07 ± 9.7E‐08	100.00		.008
	PPA	6	9.60E‐08	2.62E‐07	2.05E‐07 ± 5.94E‐08	42.30	.003	
	Bee pollen and probiotics	5	2.35E‐07	5.21E‐07	3.91E‐07 ± 1.35E‐07	80.70	.566	
	Therapeutic	5	3.03E‐07	7.31E‐07	4.60E‐07 ± 1.64E‐07	94.85	.992	
	Protective	4	2.63E‐07	6.35E‐07	4.08E‐07 ± 1.64E‐07	84.14	.756	

^a^ *p* value between each group and the control group.

^b^ *p* value between all groups.

**TABLE 2:** Pearson's positive and negative correlations between the measured parameters

Parameters	*R* (Person correlation)	*p* value	
Na^+^ (mmol/g of brain tissue ) with CK (IU/g of brain tissue)	.474*	.014	P^a^
Na^+^ (mmol/g of brain tissue) with IL‐6 (pg/g of brain tissue)	−.432*	.028	N^b^
Na^+^ (mmol/g of brain tissue) with GSH (µg/g of brain tissue)	−.737**	.001	N^b^
K^+^ (mmol/g of brain tissue) with CK (IU/g of brain tissue)	.399*	.044	P^a^
CK (IU/g of brain tissue) with GSH (µg/g of brain tissue)	−.538**	.005	N^b^
IL‐6 (pg/g of brain tissue) with GSH (µg/g of brain tissue)	.531**	.005	P^a^
GABA (pg/g of brain tissue) with Glutamate (µg/g of brain tissue)	−.446*	.022	N^b^
GABA (pg/g of brain tissue) with GABA/Glutamate ratio	.972**	.001	P^a^
Glutamate (µg/g of brain tissue) with Catalase (U/g of brain tissue)	.423*	.031	P^a^
Glutamate (µg/g of brain tissue) with GABA/Glutamate ratio	−.626**	.001	N^b^

^a^ Positive correlation.

^b^ Negative correlation.

* Correlation is significant at the .05 level.

** Correlation is significant at the .01 level.

**TABLE 3:** Analysis of receiver operating characteristics (ROC) of the parameters measured in the brain homogenate of the treated rats' pups

Parameters	Groups	AUC	Cutoff value	Sensitivity %	Specificity %	*p* value
Na^+^ (mmol/g)	PPA	0.889	0.478	83.3%	100.0%	.025
	Bee pollen and probiotics	0.533	0.414	60.0%	66.7%	.855
	Therapeutic	0.850	0.478	80.0%	100.0%	.055
	Protective	1.000	0.543	100.0%	100.0%	.011
K^+^ (mmol/g)	PPA	0.944	0.095	100.0%	83.3%	.010
	Bee pollen and probiotics	0.833	0.097	60.0%	100.0%	.068
	Therapeutic	0.700	0.086	100.0%	50.0%	.273
	Protective	0.792	0.091	100.0%	66.7%	.136
LDH (U/g)	PPA	0.611	31.779	66.7%	66.7%	.522
	Bee pollen and probiotics	0.767	27.956	100.0%	50.0%	.144
	Therapeutic	0.717	28.714	100.0%	50.0%	.235
	Protective	0.667	31.854	100.0%	66.7%	.394
CK (IU/g)	PPA	1.000	72.936	100.0%	100.0%	.004
	Bee pollen and probiotics	0.800	66.535	80.0%	83.3%	.100
	Therapeutic	0.733	51.433	100.0%	66.7%	.201
	Protective	1.000	70.229	100.0%	100.0%	.011
IL‐6 (pg/g)	PPA	0.556	563.046	66.7%	66.7%	.749
	Bee pollen and probiotics	0.667	584.677	80.0%	83.3%	.361
	Therapeutic	0.600	563.046	80.0%	66.7%	.584
	Protective	0.917	528.394	100.0%	83.3%	.033
Catalase (U/g)	PPA	0.667	20.524	83.3%	50.0%	.337
	Bee pollen and probiotics	0.667	20.341	100.0%	50.0%	.361
	Therapeutic	0.833	21.655	100.0%	66.7%	.068
	Protective	0.542	21.643	50.0%	66.7%	.831
GSH (µg/g)	PPA	0.972	16.637	100.0%	83.3%	.006
	Bee pollen and probiotics	0.517	18.535	60.0%	66.7%	.927
	Therapeutic	0.600	18.324	80.0%	66.7%	.584
	Protective	1.000	10.850	100.0%	100.0%	.011
MDA (µmol/g)	PPA	0.778	3.56E‐03	66.7%	83.3%	.109
	Bee pollen and probiotics	0.700	3.08E‐03	100.0%	50.0%	.273
	Therapeutic	0.533	3.27E‐03	60.0%	66.7%	.855
	Protective	0.562	3.27E‐03	50.0%	66.7%	.749
Glutamate (µg/g)	PPA	0.778	871.095	83.3%	83.3%	.109
	Bee pollen and probiotics	0.867	809.330	100.0%	66.7%	.045
	Therapeutic	0.567	772.265	60.0%	83.3%	.715
	Protective	0.625	805.420	75.0%	66.7%	.522
GABA (pg/g)	PPA	1.000	263.855	100.0%	100.0%	.004
	Bee pollen and probiotics	0.533	259.535	40.0%	100.0%	.855
	Therapeutic	0.733	363.676	80.0%	83.3%	.201
	Protective	0.750	354.019	75.0%	83.3%	.201
GABA/Glutamate ratio	PPA	1.000	3.20E‐07	100.0%	100.0%	.004
	Bee pollen and probiotics	0.633	3.16E‐07	40.0%	100.0%	.465
	Therapeutic	0.633	3.73E‐07	40.0%	100.0%	.465
	Protective	0.667	3.49E‐07	50.0%	100.0%	.394

## 4 DISCUSSION

Sodium (Na^+^) and Potassium (K^+^) are two vital cations, being the most abundant cations in the extracellular and the intracellular fluids, respectively. In neurons, the flow of sodium and potassium in and out of the cell through sodium–potassium pump creates an action potential (AP) (Forrest, 2014; Pohl et al., 2013).

The elevation of sodium and potassium levels in PPA‐treated rats is seen in Table 1 and Figure 2a, and that could be explained by Good (2011) who hypothesized a strong connection between low sodium levels and autism. Autistic children suffer from hyponatremia caused by diarrhea. The author attributed hyponatremia to the increased testosterone that induce the release of vasopressin which makes the kidney reabsorb water and causing a dilution in sodium levels (Córdoba et al., 2010).

Lactate dehydrogenase is a cytoplasm enzyme present in brain, is released into the blood when the brain is injured, and the rise of its serum level is usually concomitant with decrease of brain LDH relative to the degree of brain damage. In the present study, LDH did not demonstrate any significant alterations either in response to PPA neurotoxicity or the therapeutic and protective effects of combined treatments (Table 1 & Figure 2a). This is not in good agreement with the study of Al‐Orf et al. (2018), which recorded significant decrease in brain LDH as neurotoxic effect of PPA. Additionally, it is not in accordance with Al‐Osaimi et al. (2018) who reported significant improvement in brain LDH post‐treatment with bee pollen. This might be related to the differences in either the type of animal model or rout of administration of brain neurotoxic substance. The protective effect of combined bee pollen and probiotic can be easily noticed.

In Table 1 and Figure 2a, a significant elevation of CK is shown in PPA‐treated rats pups compared with control (169.45%, *p* < .001). This finding is supported by El‐Ansary et al. (2012) and could be explained by noticeable increase in activities of Ca^+2^/Mg^+2^ and Na+/K+ ATPases with association with diminishing in expression of mitochondrial electron transport chain (ETC) complexes in many brain areas in autistic children compared with control (Chauhan et al. 2011). Also, Al Mosalim et al. (2009) found elevated CK levels in Saudi autistic patients along with low ATP levels in their plasma samples.

The mixture of bee pollen and probiotic exhibited a therapeutic impact compared with protective impact, and this is in good agreement with Al‐Orf et al. (2018) and Al‐Osaimi et al. (2018) who found a decrease in CK levels after treatment with probiotic and bee pollen separately, respectively. A study showed a treatment with Protexin® lowered CK activity in birds' serum (Vahdatpour et al. 2011), as well as Hosseini et al. (2016) who demonstrated a decrease of CK activity after bee pollen intake in boiler breast muscle.

In Table 1 and Figure 2b, there is slight nonsignificant decrease of IL‐6 levels in PPA‐treated rats pups. This finding is in contrast with (El‐Ansary et al. 2012) which demonstrated increased levels of IL‐6 in brain homogenate of PPA‐treated rats. This can be attributed to the efflux of IL‐6 from brain to blood through the disrupted blood–brain barrier (Banks et al., 1995; Chen et al., 1997). This can find support through multiple studies which prove elevation of IL‐6 in plasma of autistic patients (Guloksuz et al. 2017; Inga Jácome et al. 2016; Saghazadeh et al. 2019).

In Table 1 and Figure 2b, the protective potency of the mixture was more effective compared with therapeutic, and the protective group showing *p* value at the margin of statistical significance (*p* < .075) close to being statistically significant (Dahiru, 2008). This shows the amelioration effect of the bee pollen and probiotics mixture against PPA‐induced neuroinflammation. This is in good agreement with (Aabed, Bhat, Al‐Dbass, et al., 2019; Aabed, Bhat, Moubayed, et al., 2019) which bee pollen showed a decrease in IL‐6 levels compared with PPA‐treated hamsters, as well as (Hegazi et al., 2015) whom reported the active substances of bee pollen which can act upon immunity responses. In probiotic case, *Lactobacillus plantarum* was able to reduce IL‐6 levels caused by ethanol‐induced neuroinflammation (Shukla et al., 2020), also Magistrelli et al. (2019) found that probiotics reduced IL‐6 levels in blood samples of Parkinson's disease patients.

In Table 1 and Figure 2b, PPA‐treated animals group demonstrated a significant reduction of GSH (*p* < .001) compared with control. GSH depletion in this study is in good agreement with previous studies by (El‐Ansary et al., 2012; Macfabe et al., 2007) which both reported a depletion of GSH in PPA‐treated rats, who hypothesized elevated levels of PPA could stimulate the oxidative stress in brain, the first is orally administrated while the second intraventricularly administrated along with repetitive, social, and object‐directed behaviors (MacFabe et al., 2011).

An increase of GSH levels is seen in therapeutic group (Table 1 and Figure 2b), which shows the combined effect of bee pollen and probiotics. This finding is in similar manner with a recent study which exhibited an improvement of GSH levels after probiotic and bee pollen treatment separately (Aabed, Bhat, Al‐Dbass, et al., 2019). Also a significant increase of GSH concentration is shown on a study after probiotic treatment (Al‐Orf et al., 2018). Additionally, the ameliorative effects of bee pollen can find support in the study of Al‐Osaimi et al. (2018) which manifested a therapeutic effect of bee pollen through the restoration of GSH levels in MeHg‐induced rodent model of autism.

On the other hand, in protective group (Table 1 and Figure 2b) GSH concentration is significantly reduced (29.41%, *p* < .001). This could be explained through the relation between depleted GSH level and elevated glutamate. Koga et al. (2011) reported that neurotoxicity significantly inhibits γ‐glutamyl cysteine ligase as an important enzyme in GSH cycle through which glutamate is used to synthesize GSH.

Glutamate is an anion of glutamic acid and, in CNS, it is an excitatory neurotransmitter which releases from presynaptic neurons and to postsynaptic neurons causes depolarization, of the latter, and stimulate the spread of action potentials (El‐Ansary et al., 2017; Meldrum, 2000). Glutamate have been contributed in Ca^2+^ homeostasis, developmental plasticity, and many neural physiological processes, directly and indirectly, as well as its relation with brain disease as epilepsy, stroke, Alzheimer's disease, and autism since many studies reported atypical of its signaling pathways (El‐Ansary et al., 2017; Mattson, 2008).

In Table 1 and Figure 3c, PPA‐treated rat pups showed an elevation of glutamate levels which would stimulate glutamate receptors and causing excitotoxicity and ultimately neural death. This finding is in good agreement with El‐Ansary et al. (2012) and El‐Ansary et al. (2017) who exhibited a similar elevation of glutamate levels concomitant with caspase 3 as pro‐apoptotic marker in brain homogenate of PPA acute‐treated rats.

The mixture showed a therapeutic and protective effects on normalizing glutamate levels after the excitotoxicity caused by PPA. This is also in good agreement with El‐Ansary et al. (2017) and Al‐Ghamdi et al. (2014) who reported the effectiveness of bee pollen or coenzyme Q10 as an ingredient of bee pollen in lowering glutamate levels. Also, our finding is in good agreement with El‐Ansary et al. (2018) who showed that a treatment with probiotic reduces glutamate excitotoxicity through restoring Mg^2+^ level (Stein & Glasier, 1992).

ɣ‐ aminobutyric acid, an inhibitory neurotransmitter, is a product of glutamate decarboxylation by glutamic acid decarboxylase enzyme (Rowley et al., 2012). Unlike glutamate, it prevents Ca^2+^ passage into the neurons and therefore inhibits the neural excitability (Li & Xu, 2008). It usually induces hyperpolarization of neuronal membranes, as an inhibitory signaling thus balance the depolarizing excitotoxic effect of glutamate. Aside from being an inhibitory neurotransmitter, GABA is also involved in cell's differentiation, proliferation and death, GI motility, and immune response. Its dysregulation is related to psychiatric disorders as depression and anxiety, as well as other neurological disorders like seizure and autism (Gaetz et al., 2014; Rowley et al., 2012).

In Table 1 and Figure 2c, a significant decrease of GABA levels in PPA‐treated rats pups (*p* < .005) was supported by El‐Ansary et al. (2012) in which brain homogenates of PPA‐treated rats demonstrated similar decrease in GABA levels compared with control. This is in good agreement with studies which showed a disturbance in GABAergic neurotransmission in autistic patients and that is relevant to their hyperactivity (Cochran et al., 2015; El‐Ansary & Al‐Ayadhi, 2014; Pretzsch et al., 2019).

In Table 1 and Figure 2c, the mixture showed the improvement effects of probiotics and bee pollen in ameliorating the neurotoxic effect of PPA by elevating its levels. It is supported by the ability of gut microbiota to regulate HPA axis via synthesis of neurotransmitters as GABA, and by El‐Ansary et al. (2018) who demonstrated the amelioration effect of probiotics in restoring GABA levels after PPA treatment, as well as Bravo et al. (2011) work which showed the upregulation of GABA receptors expression after indigestion of *lactobacillus*. El‐Ansary et al. (2017) and Ben Bacha et al. (2020) reported the amelioration effects of bee pollen in elevated GABA levels in brain homogenate of PPA‐treated rats and neonates of MeHg‐treated rats mothers, respectively, and this could be related to anti‐oxidant activity and neuroprotective effect of bee pollen along with high content of flavonoid which was found in brain tissue of rodents after oral gavage therefore indicating its ability to pass BBB (Franco et al., 2010; Xue et al., 2012).

Balanced ratio between GABA as inhibitory neurotransmitter and glutamate as excitatory neurotransmitter is essential in maintaining a normal neural function, since disruption of their levels would cause neurological disorders and social impairments as autistic patients (Ford et al., 2017). According to the data obtained from glutamate and GABA (Table 1 and Figure 2c), their ratio (Table 1 and Figure 3c) is significantly decreased in PPA‐treated rats pups compared with control (*p* < .005), and this is in good agreement with previous works in which they found low ratio of GABA/glutamate in PPA‐treated rats (El‐Ansary et al., 2017, 2018) as well as GABA/glutamate levels were decreased in frontal lobe of autistic patients compared with control (Harada et al., 2011). Studies have exhibited such a disturbance in GABA/glutamate in ASD patients which appears as social, memory‐related, sensory, and emotional imbalance; and this may be explained by Casanova et al. (2006) work which demonstrated changes in the number of glutamatergic and GABAergic neurons via analysis of postmortem tissues from age‐matched ASD patients (El‐Ansary & Al‐Ayadhi, 2014; Pizzarelli & Cherubini, 2011; Rosa et al., 2016). As previously mentioned how the mixture of bee pollen and probiotic was effective as protective and therapeutic in restoring the levels of glutamate and GABA, their GABA/glutamate ratio was elevated as well for the same reasons.

The significant correlations between the biochemical statuses are observed in Table 2. CK as energy replenishing marker is positively correlated with Na^+^ and K^+^; as two cations closely related to Na^+^/K^+^ ATPase, as an energy‐consuming enzyme, that is. the more active Na^+^/K^+^ ATPase, the highly active is CK to replenish the depleted ATP (Al‐Mosalim et al., 2009). However, CK is negatively correlated with GSH as oxidative stress marker and that can be elucidated by a study which observed that GSH supplementation has the ability to reduce the high levels of CK caused by impairment of plasma redox status in hypoglycemia, a risk factor in autism (Hoirisch‐Clapauch & Nardi, 2019; Jiang et al., 2007). Na^+^ is negatively correlated with IL‐6, a neuroinflammation marker. This could find support through the study of Li et al., (2014) which demonstrated the IL‐6 suppression effect in voltage‐gated sodium channel (VGSC) currents which also led to suppression of spike amplitude in rat spinal cord neurons. Likewise, Na^+^ is negatively correlated with GSH. Contrarily, Clark et al. (1996) demonstrated that the GSH depletion in the brain of rats caused by hyponatremia is recovered when Na^+^ levels are normalized in vivo.

Neuroinflammation marker (IL‐6) and oxidative stress marker (GSH) are positively correlated as expected; due to their multiple neuroprotective effects besides the regulatory role of GSH in neuronal hippocampal cells (Schmidt et al., 2005).

Glutamate as excitotoxicity marker is positively correlated with oxidative stress marker, catalase. The relation between glutamate excitotoxicity and oxidative stress is well known as elevation of the first causes an elevation of the second. However, several studies showed that catalase levels were reduced especially in brain tissue under excitotoxicity (Al‐Orf et al., 2018; Al‐Osaimi et al., 2018; Singh & Ahluwalia, 2002; Singh et al., 2003). This can be explained by the fact that if the oxidative stress level is very high or persistent, intense demolition of protein happens causing a decrease of catalase levels (by oxidative stress‐altered gene expression and/or direct oxidative damage of catalase molecules) (Al‐Ansary et al., 2013). On the other hand, the negative correlation observed between glutamate as an excitatory neurotransmitter and GABA and/or GABA/glutamate ratio as markers of inhibitory neurotransmission clearly showed the contribution of imbalanced GABA/glutamate signaling as persistent biochemical autistic features (El‐Ansary et al., 2012).

The obtained positive and negative correlations may clarify the inter‐relationships between different studied parameters as etiological mechanisms associated with neurotoxicity of the brain.

Table 3 presents ROC analysis showing AUC together with the cutoff values of the ten parameters measured for the four investigated groups. It can be noticed that CK, GABA, GABA/glutamate ratio, and GSH can be used as predictor markers of PPA neurotoxicity with AUC greater than 0.8. On the other hand, Na^+^, CK, IL‐6, GSH, and catalase demonstrated different levels of acceptable validity with different values of AUC to either therapeutic (Na^+^ and catalase) or protective (Na^+^, CK, IL‐6, and GSH) potencies to the tested bee pollen and probiotics mixture, while the rest of the parameters recorded fair predictive values. However, LDH, glutamate, and lipid peroxidation (Malondialdehyde, MDA) recorded fair predictive values toward PPA neurotoxicity as well as therapeutic and protective potency of the tested mixture.

In conclusion, bee pollen and probiotic demonstrated remarkable combined effects in ameliorating the neurotoxic effect of PPA.

## DATA AVAILABILITY STATEMENT

All data generated or analyzed during this study are included in this published article.

## CONFLICT OF INTEREST

The authors declare no conflict of interest.

## ETHICAL APPROVAL

All procedures performed in studies involving animals were in accordance with the ethical standards of the institution or practice at the Faculty of Science‐King Saud University (KSU‐SE‐19‐35).
